# Breast Cancer Knowledge and Associated Behaviors in Northern Borders, Saudi Arabia: A Cross-Sectional Study

**DOI:** 10.7759/cureus.59893

**Published:** 2024-05-08

**Authors:** Ryanh H Alanazi, Anas Fathuldeen, Malik A Hussain, Ziyad Alharbi, Layan T Almazyad, Hadeel T Alanazi, Khulud Hamed S Alshammari, Shmoukh Mushref Alruwaili, Madhawi A Alanazi, Manal S Fawzy

**Affiliations:** 1 Faculty of Medicine, Northern Border University, Arar, SAU; 2 Department of Plastic and Reconstructive Surgery, College of Medicine, University of Hail, Hail, SAU; 3 Department of Surgery, College of Medicine, Northern Border University, Arar, SAU; 4 Department of Plastic Surgery, Fakeeh College for Medical Sciences, Jeddah, SAU; 5 Unit of Medical Research and Postgraduate Studies, Faculty of Medicine, Northern Border University, Arar, SAU; 6 Department of Biochemistry, Faculty of Medicine, Northern Border University, Arar, SAU

**Keywords:** saudi women's health, risk factors, mammography screening, early detection practices, breast cancer

## Abstract

Background

Breast cancer remains a significant public health issue globally and is notably pervasive within the female population, representing a leading cause of concern. It poses a challenge across different age groups and is influenced by diverse risk factors that include genetic predispositions and various elements of lifestyle. Saudi Arabia, mirroring the global situation, has also seen its share of this disease's impact, prompting a closer look at the factors contributing to its prevalence. Educating the public and advocating for lifestyle changes are crucial steps in cancer prevention. With early-stage diagnosis and screening, many lives can potentially be saved. Our research is focused on understanding the level of awareness and preventative practices among women in the Northern Border region of Saudi Arabia. It seeks to explore the influence of familial history on knowledge and perceptions surrounding breast cancer, which could guide future educational and screening programs.

Methods

This cross-sectional study engaged 643 female participants, aged 18 and above, from the Northern Border region of Saudi Arabia upon their informed consent. Data were compiled via a structured questionnaire encompassing sociodemographic information, breast cancer knowledge, and preventive practices.

Results

The data disclosed that a significant majority (86%) recognized breast lumps as indicative of breast cancer, with 69.1% cognizant of hereditary risks. Awareness about lactation as a preventative strategy was noted in 76.7% of the participants, followed by 70.6% acknowledging the merits of a healthy diet. The study unveiled no substantial awareness disparity between individuals with or without a family history of the disease. Alarmingly, 80.4% had never sought a breast examination, and a parallel 83.7% had not undergone mammography.

Conclusion

The study sheds light on the heterogeneity in breast cancer awareness among women in Saudi Arabia's Northern Border region. Although the recognition of lumps and the preventative role of lactation is relatively high, there remains a deficit in comprehending additional symptoms, signs, and risk factors. The conspicuously low rates of breast cancer examinations and mammography underscore an urgent need for enhanced educational initiatives and a strategic push toward bolstering participation in regular cancer screenings.

## Introduction

Breast cancer, characterized by the uncontrolled proliferation of abnormal breast cells, culminates in the formation of potentially fatal invasive tumors. World Health Organization reports from 2024 delineate breast cancer as a prevalent disease affecting individuals post puberty, with a heightened incidence in advanced age. Alarmingly, in 2022, the global statistics stood at 2.3 million women diagnosed and 670,000 acceding to this condition [1]. Specifically, in Saudi Arabia, 2018 witnessed a breast cancer prevalence of 29.7% among women [2], prompting considerations that an escalation in risk factors may be propelling this upward trend [3].

Recognized risk factors encompass physiological, lifestyle, and reproductive elements, notably age, obesity, smoking, sedentariness, dietary habits, hormonal milestones, and family history [3]. The timeliness of diagnosis critically dictates outcomes; delays often result in advanced disease stages, elevated mortality, and diminished survival prospects [4,5]. Although late-stage detection is common, contributing to suboptimal prognoses, early diagnosis juxtaposes with North America's 80% five-year relative survival rates [6].

In Saudi Arabia, investigations have scrutinized women's breast cancer literacy levels and willingness to engage in breast self-examination (BSE), unearthing prevalent knowledge voids [7-11]. A startling revelation from a 2022 study indicated that only 4.2% of the participants demonstrated adequate BSE knowledge [9]. In this milieu, the necessary correlation between breast cancer awareness, self-examination skills, and consistent personal monitoring cannot be overstated. This study thereby aims to critically assess the depth of knowledge and the extent of proactive practices relating to breast cancer among the female population in Northern Border, Saudi Arabia.

## Materials and methods

Study design

This cross-sectional observational study on the female population was conducted in the Northern Borders Province of Saudi Arabia. The aim was to assess breast cancer knowledge and practices and to identify knowledge discrepancies among women with and without a family history of breast cancer. This study took place between December 2023 and April 2024.

Sampling and data collection

Data were collected using a convenience sampling method via an online, self-administered questionnaire. This type of sampling was chosen due to the logistical and time constraints often faced in our cross-sectional study and the need to reach a targeted demographic quickly and effectively. Employing the Raosoft sample size calculator (http://www.raosoft.com/samplesize.html, Raosoft, Inc., Seattle, WA) with parameters set at a 95% confidence level, a 5% margin of error, and a population of 373,577 at the 2022 census for the Northern Border area of Saudi Arabia and with 50% expected response distribution, the calculated minimum sample size was 384 women. However, we included additional participants to guarantee the adequacy and precision of the study outcomes.

Inclusion and exclusion criteria

The participants were eligible if they were (a) female residents of the Northern Border region in Saudi Arabia (our study focused on this demographic group due to the region's unique socioeconomic and cultural characteristics, which might influence breast cancer knowledge and behaviors), (b) aged 18 years or older (this age criterion was set to ensure that the participants had reached the age of majority, making them legally capable of providing consent to participate in the study without parental or guardian approval), (c) able to provide informed consent (the study participants were included if they could understand and agree to the study procedures as described in the informed consent documentation), and (d) willing to participate. Meanwhile, to avoid bias in the assessment of breast cancer knowledge and associated behaviors, individuals currently diagnosed with or undergoing treatment for breast cancer and female healthcare practitioners were excluded as their knowledge and behaviors might differ significantly from the general population due to their experience with the disease and healthcare system. Also, responses with incomplete data were excluded.

Questionnaire development and validation

A previously validated questionnaire was adapted for use [12]. It underwent evaluation and endorsement by plastic and general surgery consultants. Originally in English, the questionnaire was translated into Arabic to ensure clarity for the target demographic, with subsequent approval after a comprehensive language expert review. A pilot study with 20 participants tested the survey's comprehensibility, though these responses were excluded from the final analysis.

Distribution

The Google Forms-developed questionnaire (Google, Inc., Mountain View, CA) was disseminated across multiple social media platforms to reach diverse participants. Some of these platforms included Facebook, where we leveraged posts and targeted ads; Twitter, utilizing hashtags relevant to our research to amplify reach; and Instagram, using visually engaging posts and stories. Each platform was chosen for its unique user demographics and the potential to engage with different population subsets, ultimately engaging 643 qualified women in the study.

Survey composition

Three major sections constituted the survey, targeting (a) sociodemographic data, (b) knowledge of breast cancer, and (c) breast cancer-related practices.

Sociodemographic Information

Variables included regional residence, age groups (18-29, 30-39, 40-59, and 60+ years), nationality (Saudi or non-Saudi), education level (from illiteracy to university education), and employment status (housewife, student, and employed).

Breast Cancer Knowledge Assessment

Awareness aspects covered in the questionnaire ranged from mammography to BSE knowledge. Questions probed for familiarity with breast cancer symptoms and risk factors, including contraceptive use, hormone replacement therapy (HRT), radiation, smoking, age, and genetics. The participants also reported their primary channels of breast cancer information.

Practice and Attitude Evaluation

Practice-oriented questions explored the subjects' personal and family medical history, clinical examinations, mammograms, BSE proficiency, and openness to BSE training.

Statistical analysis

Descriptive statistics summarized participant characteristics, generating frequencies and percentages. Inferential statistical methods analyzed relationships between variables, examining how knowledge about breast cancer signs, risk factors, and preventive measures correlated with a family history of the condition. The chi-square test was applied to assess the association between the participants' knowledge and their family history, with a p-value of <0.05 indicating statistical significance. Statistical analyses were performed using the Statistical Package for Social Sciences (SPSS) software, version 27 (IBM SPSS Statistics, Armonk, NY).

Ethical considerations

Ethical approval for the study was obtained from the Local Bioethics Committee of Northern Border University (approval number: 115/13/H). The participants were informed about the study's objectives and provided consent to participate. Confidentiality and anonymity were assured.

## Results

Sociodemographic profile of the participants

Table 1 presents the sociodemographic characteristics of the study's participants. The majority (60.5%) were young adults aged between 18 and 29. Individuals within the 40-49 age range constituted 19.3% of the sample, and those aged 30-39 and 50-64 years accounted for 15.9% and 4.4%, respectively. Most respondents were Saudi nationals, representing 96.3% of the population surveyed. Regarding educational attainment, 70.1% of the participants had completed a bachelor's degree or higher. Secondary education holders were 20.8%, while postgraduate degrees and preparatory, primary, and nonliterate levels followed by 5.3%, 2.2%, 0.9%, and 0.6%, respectively. Regarding occupational status, students formed the largest subgroup, making up 46.3% of the respondents. Clerical workers succeeded this at 34.1% and housewives at 13.2%. Notably, 6.4% of the participants were employed in nonclerical roles.

**Table 1:** Sociodemographic characteristics of the studied participants (N = 643) Data are presented as frequencies (N) and proportions (%)

Variable	Categories	Frequency, N (%)
Age	18-29 years	389 (60.5%)
	30-39 years	102 (15.9%)
	40-49 years	124 (19.3%)
	50-64 years	28 (4.4%)
Nationality	Saudi	619 (96.3%)
	Non-Saudi	24 (3.7%)
Education	Illiterate	4 (0.6%)
	Primary	6 (0.9%)
	Preparatory	14 (2.2%)
	Secondary	134 (20.8%)
	Bachelor	451 (70.1%)
	Postgraduate	34 (5.3%)
Occupation	Clerk	219 (34.1%)
	Not a clerk	41 (6.4%)
	Housewife	85 (13.2%)
	Student	298 (46.3%)

**Figure 1:**
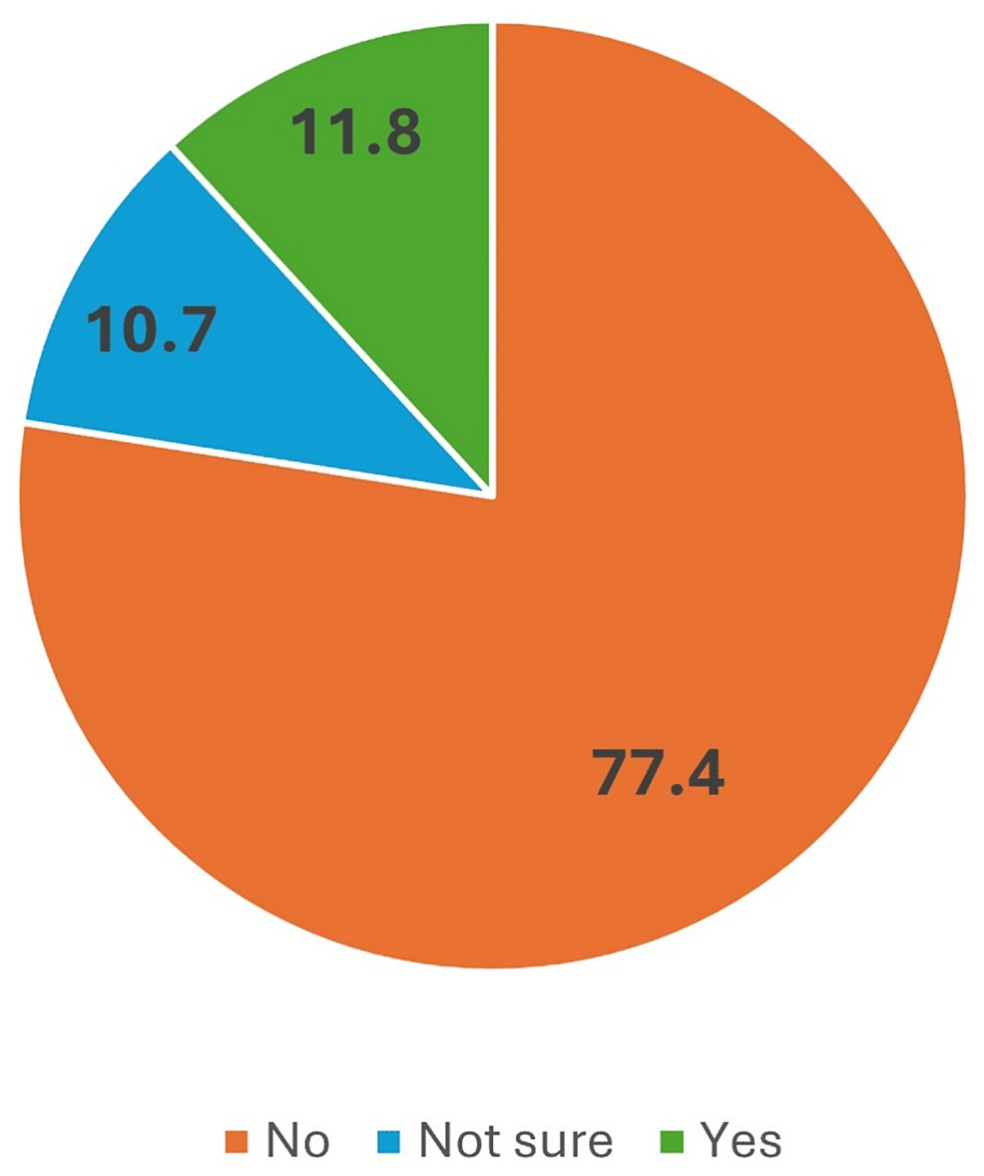
Prevalence of family history of breast cancer among the respondents Data are presented as percentages

Figure 1 shows that more than three-fourths of the respondents (77.4%) reported no family history of breast cancer. A smaller proportion of the respondents (10.7%) indicated uncertainty regarding their family history of breast cancer. A notable but smaller percentage of the respondents (11.8%) reported having a family history of breast cancer.

Awareness of signs and symptoms

The survey revealed that a worthy 86% of the participants are aware of breast lumps as an indicator of breast cancer. Nevertheless, public knowledge diminishes concerning other symptoms, with 58.6% recognizing nipple alterations, 57.4% acknowledging heaviness in the axilla, and only 53% having awareness of nipple discharge as potential signs.

Understanding of risk factors

In the context of risk factors, there is a discernible disparity in awareness. Genetic predisposition is recognized by 69.1% of the respondents, yet familiarity declines for factors such as radiation exposure (57.5%), hormone replacement therapy (HRT) (51.8%), oral contraceptive pills (OCPs) (50.7%), and smoking (48.4%). Notably, only 40.3% acknowledges age as a contributor to breast cancer risk.

Knowledge of preventive practices

Conversely, the surveyed cohort exhibited a firmer grasp of breast cancer preventive measures. A significant 76.7% of the participants understood the protective role of lactation, while 70.6% were informed about the benefits of a healthy diet, and 61% recognized the importance of regular exercise. Awareness dropped, however, regarding the preventive impact of pregnancy before age 40, with only 40.3% of the respondents citing it as a preventive factor. These insights are further detailed in Table 2.

**Table 2:** Assessment of knowledge levels on breast cancer: signs, risk factors, and preventive measures among the respondents (N = 643) Data are presented as frequencies (N) and proportions (%) HRT, hormone replacement therapy; OCPs, oral contraceptive pills

Aspect of knowledge	Categories	Frequency, N (%)
Knowledge about signs and symptoms	Nipple changes	377 (58.6%)
	Heaviness in the axilla	369 (57.4%)
	Breast lump	553 (86%)
	Nipple secretion	341 (53%)
	Change in breast size	553 (86%)
Knowledge about risk factors	HRT	333 (51.8%)
	Radiation	370 (57.5%)
	OCPs	326 (50.7%)
	Age	259 (40.3%)
	Genetics	444 (69.1%)
	Smoking	311 (48.4%)
Knowledge about preventive measures	Lactation	493 (76.7%)
	Pregnancy before 40 years	259 (40.3%)
	Diet	454 (70.6%)
	Exercise	392 (61.0%)

**Figure 2:**
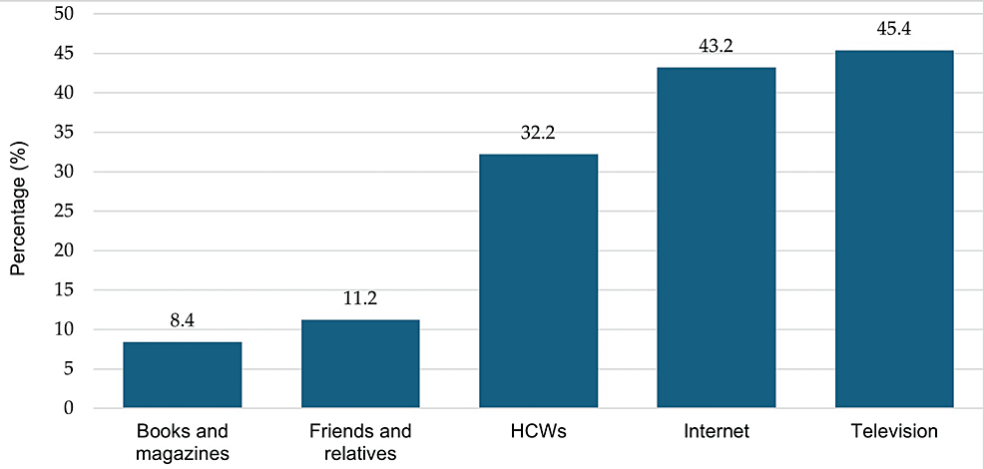
Sources of information on breast cancer awareness among the respondents (N = 643) Data are represented as percentages HCWs: healthcare workers

A significant number of the participants cited television (45.4%) and the internet (43.2%) as their primary channels for breast cancer information. Healthcare workers were also a key source, with 32.2% of the respondents seeking information from these professionals. Conversely, fewer individuals relied on friends and relatives (11.2%) or books and magazines (8.4%) for their knowledge about the disease. Further details are illustrated in Figure 2.

History of breast cancer screening

The majority of our respondents (80.4%) reported never having undergone a clinical breast cancer examination. Only 14.6% confirmed being examined, while 5% remained uncertain about their examination history. Correspondingly, 83.7% have never had a mammogram, compared to the 11% who have. An additional 5.3% could not recall if they had undergone this screening.

When it comes to self-conducted breast examinations at home, 53% of the participants indicated that they had never performed one, and 39.5% had. Uncertainty was expressed by 7.5% of the respondents. Of the women who had not performed self-examinations (n = 341), 63.6% showed an interest in learning the procedure. Meanwhile, 25.5% were not inclined to learn, with 10.9% undecided. These findings are tabulated in Table 3.

**Table 3:** Assessment of breast cancer examination history and intention to learn self-examination techniques among the respondents (N = 643) Data are presented as frequencies (N) and proportions (%)

Studied variables	Categories	Frequency (%)
Have you ever had a breast cancer examination?	No	517 (80.4%)
	Not sure	32 (5%)
	Yes	94 (14.6%)
Have you ever had a breast mammogram?	No	538 (83.7%)
	Not sure	34 (5.3%)
	Yes	71 (11%)
Have you ever had a breast cancer examination at home?	No	341 (53%)
	Not sure	48 (7.5%)
	Yes	254 (39.5%)
Do you have the intention to learn how to conduct breast examination (n = 341)?	No	87 (25.5%)
	Not sure	37 (10.9%)
	Yes	217 (63.6%)

The study findings indicate no significant variation in the awareness of breast cancer between the participants with and without a family history of the disease, as detailed in Table 4.

**Table 4:** Association between the knowledge of breast cancer and family history among the respondents (N = 643) Data are presented as percentages. A chi-square test was applied. Significance was set at a p-value of <0.05 HRT, hormone replacement therapy; OCPs, oral contraceptive pills

Knowledge		Family history of breast cancer				P-value
		No		Yes		
		N	%	N	%	
Nipple changes	No	240	88.2	32	11.7	0.567
	Yes	340	86.7	52	13.3	
Heaviness in the axilla	No	247	87.3	36	12.7	0.963
	Yes	333	87.4	48	12.6	
Breast lump	No	86	92.5	7	7.5	0.109
	Yes	494	86.5	77	13.5	
Nipple secretion	No	269	86.5	42	13.5	0.534
	Yes	311	88.1	42	11.9	
Change in breast size	No	86	92.5	7	7.5	0.109
	Yes	494	86.5	77	13.5	
HRT	No	276	85.7	46	14.3	0.219
	Yes	304	88.9	38	11.1	
Radiation	No	239	86.6	37	13.4	0.621
	Yes	341	87.9	47	12.1	
OCPs	No	290	88.4	38	11.6	0.415
	Yes	290	86.3	46	13.7	
Age	No	342	86.4	54	13.6	0.353
	Yes	238	88.8	30	11.2	
Genetics	No	178	86.4	28	13.6	0.624
	Yes	402	87.8	56	12.2	
Smoking	No	298	87.4	43	12.6	0.974
	Yes	282	87.3	41	12.7	
Lactation	No	139	89.1	17	10.9	0.451
	Yes	441	86.8	67	13.2	
Pregnancy before 40 years	No	339	85.4	58	14.6	0.064
	Yes	241	90.3	26	9.7	
Diet	No	179	90.4	19	9.6	0.123
	Yes	401	86.1	65	13.9	
Exercises	No	231	88.5	30	11.5	0.471
	Yes	349	86.6	54	13.4	

## Discussion

Breast cancer remains one of the most common malignancies worldwide, manifesting a considerable prevalence of 29.7% in Saudi Arabia [2,13]. Our study delved into the knowledge and associated behaviors regarding breast cancer among women in Saudi Arabia's Northern Border region, aiming to quantify their level of understanding and practices concerning this disease.

A pivotal finding was the high level of awareness of breast lumps as an indicator of breast cancer, with 86% of the respondents recognizing this sign. This finding is consistent with previous studies that have shown that physical symptoms, particularly breast lumps, are often the most recognized sign of breast cancer among women due to ongoing public health campaigns emphasizing self-examination and early detection [14-16].

Moreover, the recognition of the protective role of lactation against breast cancer by 76.7% of our respondents aligns with scientific evidence that lactation reduces the risk of breast cancer, likely due to hormonal changes during breastfeeding that delay the return of menstrual periods, thus reducing lifetime exposure to hormones such as estrogen, which can promote breast cancer cell growth [17].

The finding that awareness levels were not significantly different between those with a family history of breast cancer and those without could suggests that personal experiences with the disease do not necessarily translate into improved knowledge. This finding was similar to an earlier study by Bird et al. [18], who found that the average knowledge score of women with a family history of breast cancer and those without was comparable. This could be due to the psychological impact of cancer in the family, where denial or fear may impede the acquisition of knowledge, or it might indicate a general success of widespread educational messages reaching both groups equally. However, it underscores the importance of targeted educational interventions for those with a family history, as they constitute a high-risk group for developing the disease [19].

When considering risk factors aside from genetics, such as age, BMI, tobacco usage, physical activity, diet, hormonal factors, and breast density, knowledge was variable. For instance, the awareness of the implications of radiation exposure, HRT, and OCPs fluctuated between 50.7% and 57.5%. These results are in line with recent research from the Qassim region [20] and Riyadh City [21], although a study from Al-Jouf reported lower awareness of HRT as a risk factor [22].

Our participants' recognition of smoking and age as risk factors, cited at 48.4% and 40.3%, respectively, contrasted with the higher knowledge levels observed in the Al-Jouf province study [22]. Moreover, the ability to identify breast lumps significantly varied when compared to studies executed in the Qassim [20] and Eastern regions [16], suggesting regional differences in breast cancer knowledge. This regional variation can be due to several factors, including differing sample sizes; variations in demographics such as age, educational level, and socioeconomic status; differences in access to healthcare services; the cultural attitudes toward health and breast cancer; the presence, frequency, and effectiveness of public health campaigns; and education/screening programs about breast cancer, which can vary significantly between regions and may influence knowledge and self-examination practices.

Interestingly, a substantial number of women in our study sourced their information from television and the internet, which resonates with findings from Hail [23], Qassim [24], Jeddah [25], and Abha [12]. This trend underscores the influence of media on public health awareness.

Regarding preventive measures, lactation was widely recognized as beneficial, paralleling other research underscoring its importance [26]. The core of this finding may relate to the religious and cultural belief that recommends and encourages breastfeeding [27].

Additionally, the links between diet, exercise, and breast cancer risk were acknowledged by a majority of our participants, reflecting guidance from authoritative bodies such as the American Society of Clinical Oncology [26,28]. However, knowledge regarding the preventive effect of pregnancy before age 40 was less known, with under half of the respondents aware of this factor.

Regarding screening behaviors, an overwhelming 80% had not undergone a clinical breast examination, and 83.7% had never received a mammogram, echoing low screening rates in line with other reports with similar cultural contexts [29,30]. These figures may correlate with the 53% of our respondents who have never practiced at-home breast examinations. Possible explanations for this statistic may include limited awareness about the importance of self-examinations, cultural beliefs affecting health practices, barriers to accessing healthcare services, or varying perceptions of personal risk among the target population [29-34]. By considering these factors, future studies should provide a more comprehensive understanding of the underlying reasons for the current statistics.

The willingness to learn about self-examination among the participants who have never performed it suggests an opportunity for educational interventions. Our findings highlight the critical need for continued public health efforts to improve knowledge and encourage proactive breast health behaviors. Comprehensive education efforts must leverage various channels, including media, academic institutions, and public spaces such as markets and healthcare waiting rooms, to broaden their reach. Collaborations with the Ministries of Health and Education must be strengthened to amplify the message of breast cancer awareness and to bolster early detection and screening campaigns. Through concerted efforts, we can aspire to increase the overall knowledge of breast cancer and improve outcomes through proactive health practices.

Although our study is the first in the Northern Border region of Saudi Arabia to conduct such a survey and to use a pre-validated tool to collect the specified data, potentially yielding valuable insights, it is not without limitations. The study's cross-sectional nature restricts our ability to infer causation, and the reliance on self-reporting through questionnaires may introduce response biases as only people with internet access and/or social media presence will respond to this survey. Also, using the convenience sampling method for participant recruitment may introduce certain biases, such as "selection" or "nonresponse" biases, that should be considered in future studies.

## Conclusions

This study highlights a spectrum of awareness and engagement in breast health behaviors among women in Saudi Arabia's Northern Border region. The participants displayed a commendable understanding of certain breast cancer signs, such as the presence of lumps, and recognized lactation as a preventive measure against the disease. Despite these positives, there are notable knowledge deficits concerning additional signs, symptoms, risk factors, and preventive practices.

Alarmingly, the majority of study participants have not engaged in either clinical breast examinations or mammography. These findings underscore an urgent need for enhanced health education initiatives tailored to women. There is a critical mandate to motivate regular participation in breast cancer screenings and to dispel myths surrounding mammography, particularly among those with a familial predisposition to the condition, who would benefit from annual screenings.
